# A Whole-Brain Model of the Aging Brain During Slow Wave Sleep

**DOI:** 10.1523/ENEURO.0180-24.2024

**Published:** 2024-11-05

**Authors:** Eleonora Lupi, Gabriele Di Antonio, Marianna Angiolelli, Maria Sacha, Mehmet Alihan Kayabas, Nicola Alboré, Riccardo Leone, Karim El Kanbi, Alain Destexhe, Jan Fousek

**Affiliations:** ^1^Department of Brain and Behavioral Sciences, University of Pavia, Pavia 27100, Italy; ^2^Research Center “Enrico Fermi”, Rome 00184, Italy; ^3^“Roma Tre” University of Rome, Rome 00146, Italy; ^4^Natl. Center for Radiation Protection and Computational Physics, Istituto Superiore di Sanità, Rome 00161, Italy; ^5^Department of Engineering, Università Campus Bio-Medico di Roma, Rome 00128, Italy; ^6^ Institute of Neuroscience (NeuroPSI), Paris-Saclay University, Centre National de la Recherche Scientifique (CNRS), Saclay 91400, France; ^7^Univ Rennes, INSERM, Rennes 35043, France; ^8^“Tor Vergata” University of Rome, Rome 00133, Italy; ^9^Faculty of Medicine, University of Bonn, Bonn 53115, Germany; ^10^Computational Neurology Group, Ruhr University Bochum, Bochum 44801, Germany; ^11^Deutsches Zentrum für Neurodegenerative Erkrankungen (DZNE), Bonn 53127, Germany; ^12^DREEM, Paris 75003, France; ^13^Central European Institute of Technology (CEITEC), Masaryk University, Brno 62500, Czech Republic

## Abstract

Age-related brain changes affect sleep and are reflected in properties of sleep slow-waves, however, the precise mechanisms behind these changes are still not completely understood. Here, we adapt a previously established whole-brain model relating structural connectivity changes to resting state dynamics, and extend it to a slow-wave sleep brain state. In particular, starting from a representative connectome at the beginning of the aging trajectory, we have gradually reduced the inter-hemispheric connections, and simulated sleep-like slow-wave activity. We show that the main empirically observed trends, namely a decrease in duration and increase in variability of the slow waves are captured by the model. Furthermore, comparing the simulated EEG activity to the source signals, we suggest that the empirically observed decrease in amplitude of the slow waves is caused by the decrease in synchrony between brain regions.

## Graphical abstract

**Figure:**
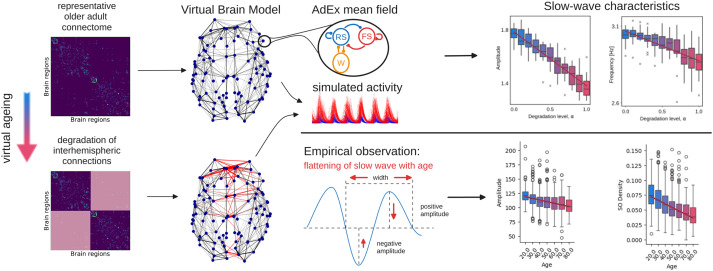
Visual Abstract

## Significance Statement

Aging is characterized by changes in slow wave (SW) sleep features, yet the precise mechanisms driving these alterations remain elusive. Employing a connectome-based model, we implement the established age-related reductions in inter-hemispheric connectivity, successfully replicating the SW changes in the simulated activity. Our simulation of EEG activity also suggests that observed decreases in SW amplitude stems from diminished synchrony between brain regions. Our results support the notion that alterations in SW characteristics result from reductions in cortical excitatory drive—here facilitated by the inter-hemispheric connections. Our model serves as a robust foundation for extensions to population studies and interventional work in animal models of aging aimed at disentangling the contributions of network alterations, changes to local neural mass properties, and neuromodulation.

## Introduction

Slow waves (SW) are neural oscillations occurring during non-rapid eye movement (NREM) sleep that are characterized by a phase of hyperpolarization (down period), during which cortical neurons are silent, and by a phase of depolarization, in which intense neuronal firing occurs (up period) (Steriade et al., 1993). SW characteristics vary across the lifespan, changing in both healthy (Van Cauter et al., 2000; Landolt and Borbély, 2001; Mander et al., 2017) and pathological aging. Compared to young subjects, older individuals show a lower percentage of deep SW sleep (Van Cauter et al., 2000; Landolt and Borbély, 2001; Mander et al., 2017), lower SW density and amplitude, especially at the beginning and end of the night (El Kanbi et al., 2023), and both longer SW positive and negative phase duration, especially in prefrontal/frontal brain areas (Carrier et al., 2011). Furthermore, in a recent study of more than 2000 individuals, El Kanbi et al. (2023) also found that slow-wave inducing auditory stimulation during N3 (Debellemaniere et al., 2018) were much less successful in older subjects compared to younger ones. Although these descriptive changes of SW characteristics with aging are well known in the literature, the exact mechanisms linking aging to alterations in SW are still not fully elucidated.

Aging is associated with many structural brain alterations involving both the gray (Giorgio et al., 2010; Bethlehem et al., 2022) and white matter (Antonenko and Flöel, 2014; Damoiseaux, 2017; Puxeddu et al., 2020; Lavanga et al., 2023a) that might be implicated in the alteration of SW. Previous studies showed a reduction in cortical gray matter occurring across the lifespan (Giorgio et al., 2010; Bethlehem et al., 2022) and this has already been linked to alterations in SW (Dubé et al., 2015). Reduced cortical thickness in regions that are normally involved in SW generation (e.g., insula, superior temporal, parietal, middle frontal) mediated the negative effects of aging on SW density, whereas reduced SW amplitude was associated with reduced cortical thickness in middle frontal, medial prefrontal, and medial posterior regions (Dubé et al., 2015). Another prominent aspect of age-related structural changes is the reduction in white matter connections (Antonenko and Flöel, 2014; Damoiseaux, 2017) which was shown to occur especially for inter-hemispheric connections (Puxeddu et al., 2020; Lavanga et al., 2023a). Inter-hemispheric connections have a prominent influence on aspects of coordinated neural dynamics both during awake resting state activity (Lavanga et al., 2023a), and during sleep (Avvenuti et al., 2020). In the awake state, reductions in inter-hemispheric connectivity have been linked to a reduction in functional dynamical flexibility (Lavanga et al., 2023a). During sleep, a previous study on callosotomized patients with drug-resistant epilepsy (Avvenuti et al., 2020) reported that SW displayed a significantly reduced probability of cross-hemispheric propagation and a stronger interhemispheric asymmetry compared to controls (Avvenuti et al., 2020). Nevertheless, if reduced inter-hemispheric connectivity might play a role in shaping SW characteristics during normal aging has not yet been investigated.

**Figure 1.**
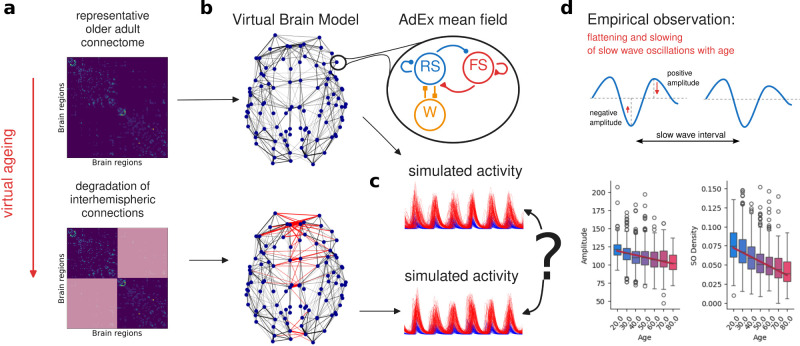
Overview of the approach. ***a***, The age-related deterioration of the interhemispheric connections is captured as a gradual decrease in the respective elements in the structural connectivity (SC) matrix representative of an older adult. ***b***, Virtual Brain model is constructed from the SCs along the virtual aging trajectory using the AdEx mean field model to govern the nodes’ dynamics. ***c***, The brain network model is used to simulate the sleep-like activity both on the source-level and on the EEG sensors, which is then compared across the virtual aging trajectory and against the empirical data with respect to the selected data features. ***d***, Empirical observations of interest (El Kanbi et al., 2023): flattening of the slow oscillations (decrease in amplitude) and decrease in density (fewer SO per time unit).

Connectome-based whole-brain modeling frameworks, such as The Virtual Brain (TVB) (Sanz-Leon et al., 2015; Schirner et al., 2022), can create personalized virtual brain models (Ghosh et al., 2008; Deco and Corbetta, 2011), that can integrate individual structural brain imaging data and, based on the mean field model (Abbott and van Vreeswijk, 1993; Treves, 1993; Brunel and Hakim, 1999; Knight, 2000; Mattia and Del Giudice, 2002) governing the dynamics of each node of the network, capture the characteristics of resting-state (Lavanga et al., 2023a) or SW dynamics (Goldman et al., 2022). Using a spiking model of adaptive exponential integrate and fire (AdEx) neurons, El Kanbi et al. (2023) suggest that the reduction of the excitatory drive might be the mechanism behind the observed SW changes on the level of individual neuronal populations. Here, we aim to investigate to which degree this reduction of excitatory drive might be related to the reduction of inter-hemispheric connectivity. In particular, by using the AdEx mean field model (Di Volo et al., 2019) and structural connectivity matrices representative of the participants of the 1000BRAINS study (Caspers et al., 2014) at the beginning of the aging trajectory, we recapitulate the main aging-associated alterations of EEG slow-wave recordings (El Kanbi et al., 2023) by manipulating the underlying structural connectivity (Lavanga et al., 2023a) (i.e., by reducing the connection strengths of inter-hemispheric tracts, Fig. 1). Then, we investigate the changes of the slow-wave characteristics in the model along the virtual ageing trajectory on the source level, and relate these to the hypothesized decrease of the excitatory drive and changes in synchrony. Through this simulation-based approach, we aim to gain insights into the underlying mechanisms linking aging, inter-hemispheric connectivity, and slow wave dynamics.

## Methods

### Structural connectivity

An anonymized connectome was drawn from an embedding of structural connectivity matrices of the youngest subjects of the 1000BRAINS project (Caspers and Schreiber, 2021; Lavanga et al., 2023b). The original connectomes resulted from a pipeline described in detail in Caspers et al. (2014). In short, for each participant, a T1-weighted and diffusion-weighted MRI images were obtained. The T1 scan (MPRAGE, 176 slices, TR = 2.25 s, TE = 3.03 ms, TI = 900 ms, FoV = 256 × 256 mm^2^, flip angle = 9°, resolution 1 mm isotropic) was used to extract cortical grey matter masks, which were divided into 100 regions using the Schaefer parcellation scheme (17-Networks, Schaefer et al., 2018). The diffusion MRI (two *b*-value scans b = 1,000 s/mm^2^ (EPI, TR = 6.3 s, TE = 81 ms, 7 b0-images and 60 DW volumes) and b = 2, 700 s/mm^2^ (EPI, TR = 8 s, TE = 112 ms, 13 b0-images and 120 DW volumes)) were registered to the T1 images, and used to generate streamlines between the respective regions defined by the parcellation, resulting in a 100 × 100 connectivity matrix. The representative connectome at the start of the aging trajectory was derived from the pseudonymized dataset (Caspers and Schreiber, 2021, N = 645, age 55–63 years).

The representative connectome for the subjects at the start of the aging trajectory was computed from 220 connectomes of subjects in the age group of 55–63 years. The connectomes were linearized and stacked resulting in a 220 × 100^2^ matrix *W*. The matrix *W* was then factorized using singular value decomposition giving *W* = *USV*^*H*^. The first 33 components were sufficient to eliminate any confusion when the connectomes were projected back into this space, so we have set the dimension of the generative distribution constructed in the next step to *d* = 33. The matrix *U* can be cast into a multivariate normal distribution $U_{N}=(\mu ,\Sigma )$ such that means are $\mu_{i}={\mathrm{avg}}_{\;j,j<d}\;(U_{ij})$ and the covariance $\Sigma =\mathrm{cov}\;(U_{ij,j<d}^{T})$. A sample *U*′ drawn from the *U*_*N*_ is then converted to a connectome *W*′ by computing $W^{\prime }=U^{\prime }\mathrm{diag}\;S_{ij,j<d}V_{ij,j<d}^{T}$ and devectorizing *W*′ back to a 100 × 100 matrix.

### Virtually aged structural connectome

The structural connectome was virtually aged (Lavanga et al., 2023a) by gradually decreasing the interhemispheric connections. In particular, given the weighted connectivity matrix *W* and the matrix $M^{\alpha }$ such that $M_{ij}^{\alpha }=1$ for intra-hemispheric connections, and $M_{ij}^{\alpha }=1-\alpha$ for inter-hemispheric connections, the gradually aged connectome was computed as element-wise product $W_{\alpha }=W⊙M_{\alpha }$ for *α* ∈ [0, 6] with a step of 0.1, resulting in 7 samples along the virtual aging trajectory. Here, the *α* = 0 signifies an unaltered connectivity matrix, while *α* = 0.6 corresponds to the highest reduction observed in the elderly subjects (Lavanga et al., 2023a).

### Modeling

The Adaptive Exponential Integrate-and-Fire (AdEx) model is a biologically realistic neuron model that has been widely used to simulate the electrical activity of neurons (Brette and Gerstner, 2005). It is an extension of the classic integrate-and-fire model, incorporating both an exponential spike mechanism and an adaptation process. The mean field equations for the AdEx model includes conductance-based synaptic interactions and accounts for adaptation, leading to the following set of equations (Zerlaut et al., 2018; Di Volo et al., 2019; Goldman et al., 2022):

$$\begin{array}{cc} & T\frac{\partial \nu_{\mu }}{\partial t}=(F_{\mu }-\nu_{\mu })+\frac{1}{2}\frac{\partial^{2}F_{\mu }}{\partial \lambda \partial \eta }c_{\lambda \eta },\\ & T\frac{\partial c_{\lambda \eta }}{\partial t}=\delta_{\lambda \eta }\frac{F_{\lambda }(1/T-F_{\eta })}{N_{\lambda }}+(F_{\lambda }-\nu_{\lambda })(F_{\eta }-\nu_{\eta })+\frac{\partial F_{\lambda }}{\partial \nu_{\mu }}c_{\eta \mu }+\frac{\partial F_{\eta }}{\partial \nu_{\mu }}c_{\lambda \mu }-2c_{\lambda \eta },\\ & \frac{\partial W_{\mu }}{\partial t}=-\frac{W_{\mu }}{\tau_{w\mu }}+b_{\mu }\nu_{\mu }+\frac{a_{\mu }\left[\mu_{V}(\nu_{e},\nu_{i},W_{\mu })-E_{L_{\mu }}\right]}{\tau_{w\mu }}, \end{array}$$

where *ν*_*μ*_ is the average firing rate of population *μ* = {*e*, *i*} (excitatory or inhibitory), $F_{\mu }=F_{\mu }(\nu_{e}+\nu_{e}^{in},\nu_{i}+\nu_{i}^{in},W_{\mu })$ is the transfer function, $\nu_{e}^{in}$ and $\nu_{i}^{in}$ are the inputs to the excitatory and inhibitory populations, *c*_*λη*_ is the covariance between populations *λ* and *η* and *W* is a population adaptation variable (Di Volo et al., 2019). The mean field model can accurately predict the average spontaneous activity levels in asynchronous irregular regimes, capture the transient temporal response of the network to complex external inputs, and quantitatively describe regimes where high–and low-activity states alternate (up/down state dynamics).

The previous equations can be extended to describe large networks of interconnected brain regions, with each region being described by a mean-field model and the connectivity derived from human tractography (structural connectivity). In addtion, such network can be driven by noise to produce spontaneous activity. In particular, the network input enters at node *k* at time *t* by expanding the excitatory input term $\nu_{e}^{in}$ as

$$\nu_{\mu }^{in}(k,t)=G_{\mu }\sum_{j}C_{kj}\nu_{e}(\;j,t-D_{k,j})+wOU(t{)}_{k},$$

where $G_{\mu }$ is a scaling factor, *C*_*kj*_ is the connectivity matrix, *D*_*k*,*j*_ is the propagation delay, *w* is the noise scaling factor, and *OU*(*t*)_*k*_ is the noise drive defined as Ornstein-Uhlenbeck process:

$$T_{OU}dOU=-OUdt+dW_{t},$$

where *T*_*OU*_ is time constant and d*W*_*t*_ is an increment of a Wiener process. The network input is scaled differently for the inhibitory and excitatory populations given the *S*_*i*_ constant, such that *G*_*e*_ = *S*_*i*_ *G*. The parameters of the model were adapted from Goldman et al. (2022) and are listed in Table 1.

**Table 1.** AdEx mean field model parameters

(a) Scalar parameters of the AdEx mean field model					
parameter	value	parameter	value	parameter	value
*C*_*m*_	200.0pF	*S*_*i*_	1.2	*g*	0.2
$E_{L_{e}}$	−63.0 mV	*T*	40.0 ms	*g*_*L*_	10.0 nS
$E_{L_{i}}$	−65.0 mV	*a*_*e*_	0.0 nS	$p_{{\text{con}}_{e}}$	0.05
*E*_*e*_	0.0 mV	*a*_*i*_	0.0 nS	$p_{{\text{con}}_{i}}$	0.05
*E*_*i*_	−80.0 mV	*b*_*e*_	60.0 pA	*T*_OU_	5.0 ms
$K_{{\text{ext}}_{e}}$	400	*b*_*i*_	0.0 pA	*τ*_*e*_	5.0 ms
$K_{{\text{ext}}_{i}}$	0	$\nu_{e}^{ex}$	0.315e−3 kHz	*τ*_*i*_	5.0 ms
*N*_tot_	10,000	$\nu_{e}^{in}$	0.000 kHz	$\tau_{w_{e}}$	500.0 ms
*Q*_*e*_	1.5 nS	$\nu_{i}^{ex}$	0.315e−3 kHz	$\tau_{w_{i}}$	1.0 ms
*Q*_*i*_	5.0 nS	$\nu_{i}^{in}$	0.000 kHz	noise weight	1e−4

(b) Values for the fitted polynomials *P* of the transfer function $F_{\mu }$. See Di Volo et al. (2019) for detailed description. Values are given in V										
cell type	*P*_0_	$P_{\mu_{V}}$	$P_{\sigma_{V}}$	$P_{\tau_{V}}$	$P_{\mu_{V}^{2}}$	$P_{\sigma_{V}^{2}}$	$P_{\tau_{V}^{2}}$	$P_{\mu_{V}\sigma_{V}}$	$P_{\mu_{V}\tau_{V}}$	$P_{\sigma_{V}\tau_{V}}$
excitatory	−0.0498	0.00506	−0.025	0.0014	−0.00041	0.0105	−0.036	0.0074	0.0012	−0.0407
inhibitory	−0.0514	0.004	−0.0083	0.0002	−0.0005	0.0014	−0.0146	0.0045	0.0028	−0.0153

For the following analyses, 30 s of data were simulated and the first initial transient of 2 s was discarded before further processing.

### EEG

The EEG observer model complements the generative model of the brain dynamics (that is the networked AdEx model) in order to provide simulated EEG signals. Here, four virtual electrodes were positioned on the forehead to simulate the placement of headband devices commonly used in sleep studie s, i.e., the Dreem Headband (Arnal et al., 2020). A detailed view of the electrodes placement is presented in Extended Data Figure 2-3. Given the small number of electrodes and coarse-grained spatial resolution of the dynamical model, a simplified forward solution ommiting the conductivities and dipole orientation was employed (Sarvas, 1987). The elements of the leadfield matrix *L* prescribing the contribution of each of the brain network nodes to the sensor-level signals, were calculated as follows:

$$L_{ij}=1/d_{ij}^{2}$$

where *d*_*ij*_ is the euclidean distance between brain region *i* the electrode *j*. The EEG signals are then derived as the dot product between the lead field matrix and the output of the AdEx model.

### Analysis

#### Detection of up- and down-states

**Figure 2.**
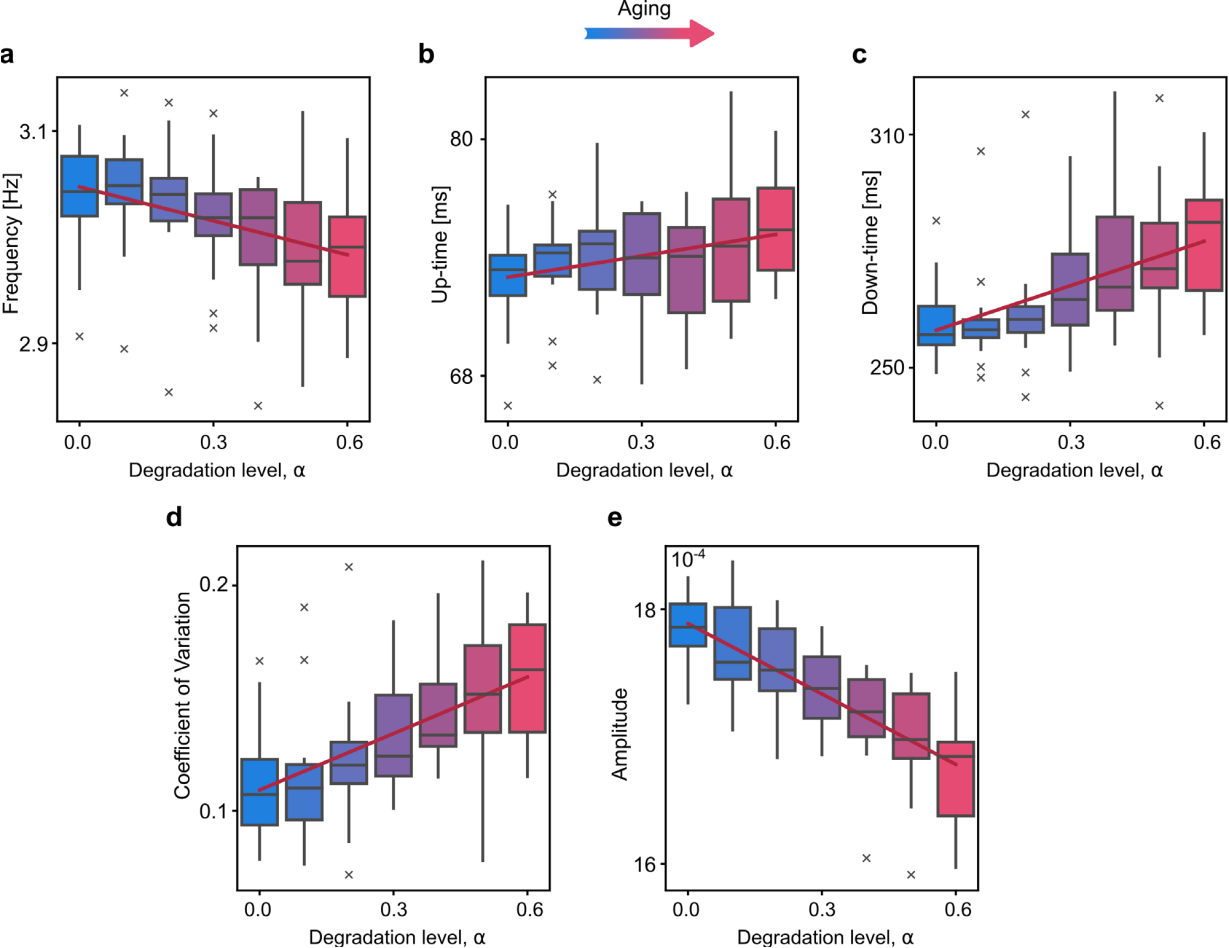
Slow oscillation (SO) changes along the virtual aging trajectory in the simulated EEG data. A value of 50 ms is taken for the spike temporal length and 0.5 for the amplitude threshold. The boxplots represent the variability of the average network characteristics for 16 different realizations of the OU process. The following effects are observed: Decrease in SO frequency ***a***, Slight increase in up-state duration ***b***, Increase in down-state duration ***c***, Increase in coefficient of variation (CV) of the SO ***d***, Decrease in amplitude of the SO ***e***, Extended Data Figure 2-1 provides the statistical evaluation of the differences. Extended Data Figure 2-2 details the robustness of the results with respect to the choice of the up-state threshold. Extended Data Figure 2-3 shows the position of the EEG electrodes.

The dynamics of the model in the sleep-like regime exhibits regular slow-wave dynamics, characterized by alternating periods of high and low activity (up- and down-states, Goldman et al., 2022). In order to quantify this dynamical pattern, we applied the following steps to identify and evaluate the up- and down-states in both the EEG and source-level time-series. The simulated EEG time-series was first normalized in the [0 − 1] range and then binarized using a threshold of 0.5 (see Extended Data Fig. 5-2). Up-states were defined as having higher activity than the threshold and down-states as having lower activity than the threshold, respectively. Then, the start and length of each up or down state were identified, and only cycles where both states lasted at least 50 milliseconds were considered for further analysis. Same procedure was also applied on the source-level time-series, with the binarization threshold of 0.3, and duration threshold of 40 milliseconds. A detailed view of the behavior for various time and amplitude thresholds for the source-level signals is presented in Extended Data Figure 3-1, and for the simulated EEG in Figure 2-2. The particular values of the thresholds were chosen to avoid misinterpreting noise-driven fluctuation in the down-state (threshold too low), and missing lower-amplitude up-states (threshold too high).

#### Slow wave characterization

To evaluate the effects of aging on the SW properties, we particularly focused on the following variables: average SW frequency, average up and down-state duration, the coefficient of variance of the SW frequency and synchrony. Each of these is further detailed below. First, the total duration of each up-down cycle was extracted as the time interval from the first point above the selected threshold to the last point recorded before the onset of a new up-state. The corresponding frequency for each SW was taken as the inverse of the duration, then the mean and standard deviation were computed based on the distribution of these frequency values, and the coefficient of variation (CV) was calculated as the ratio of the standard deviation to the mean (following El Kanbi et al., 2023).

To evaluate the effect of aging on the simulated SW EEG sleep-like activity, we focused on key parameters, including average frequency of the slow oscillations (SO), average up and down-state duration, CV of the SO and average amplitude of the SO. The data features were calculated for the EEG sensor time-series in the same way as described above for the source-level signals. Results are reported as averages across the simulated EEG channels.

On the source level, we report both the averages across all nodes of the brain network, and for the individual nodes (in a form of a spatial map), in order to account for the potential differential effects of aging across the network nodes. We also explored how the change through the synthetic aging in the above-mentioned features of the slow waves relates to the graph-theoretical measures of node significance in the structural network (node strength and eigenvector centrality). Briefly, node strength is quantified as the aggregate of the weights of all edges connected to a node, offering a measure of its significance in a weighted network. Eigenvector centrality, on the other hand, assigns scores to nodes relative to the quality of their connections, where links to nodes with high scores are more important in determining a node’s score than links to nodes with lower scores. Consequently, a node with high eigenvector centrality indicates that many of its connections are to other nodes that also have high eigenvector scores.

#### Phase synchrony

In order to characterize the phase synchrony between the different nodes, the phase locking value (PLV) was calculated. PLV was originally introduced by Lachaux et al. (1999), and it was previously used in the analysis of resting state connectivity in magnetoencephalography (MEG) (Ghuman et al., 2011), as it provides a tool to analyse temporal relationships between two neural signals without considering the amplitude of the signal. PLV expresses the absolute value of the mean phase difference between two signals and can be described as follows:

$$\text{PLV}=\left|\frac{1}{N}\sum_{t=1}^{N}e^{i(\phi_{i}(t)-\phi_{j}(t))}\right|,$$

where *i* and *j* the indices of nodes, *ϕ*(*t*) the phase of the time series extracted by the Hilbert transform at time *t* and *N* is the total number of time points, so that the metric was computed for each pair of node *i*, *j* and averaged over time. PLV values range between 0 and 1 for random and fixed phase relationships, respectively. Thus, higher values describe higher degree of synchrony. In our case, we apply PLV at the source-level of the simulated data—that is directly to the time-series of the state variables of the AdEx model in each of the nodes—hence avoiding any volume conduction biases common in EEG applications (Stam et al., 2007).

The metric was calculated by definition for each pair of nodes, for the two types of populations (excitatory and inhibitory) resulting in a nodes × nodes symmetric matrix *M*. The mean of the matrix excluding the diagonal elements was averaged over all trials to provide an estimation of whole-brain synchrony across different degrees of virtual aging. The intra- and inter-hemispheric synchrony (per hemisphere) was also explored separately, by averaging over the relevant submatrices of *M* (top-right for interhemispheric, top-left for right intrahemispheric and bottom-right for left interhemispheric synchrony). Additionally, the synchrony of frontal brain regions was calculated (see Table 2 and Fig. 5-1 for details on the regions), motivated by the frontal position of the electrodes in the experimental data (El Kanbi et al., 2023).

**Table 2.** Listing of the frontal areas included in the frontal areas synchrony exploration in Figure 5

Hemisphere	Network	Acronym
LH	LimbicB	OFC
LH	ContA	PFCl
LH	ContB	PFClv
LH	DefaultA	PFCm
LH	DefaultB	PFCv
LH	DefaultB	PFCv
RH	SalVentAttnB	PFCl
RH	LimbicB	OFC
RH	ContA	PFCl
RH	ContB	PFClv
RH	DefaultA	PFCm
RH	DefaultB	PFCv
RH	DefaultB	PFCv

#### Code accessibility

Codes implementing the simulation and analysis steps described above are publicly available as Extended Data 1 and in the following repository https://gitlab.ebrains.eu/fousekjan/vab-sleep.

10.1523/ENEURO.0180-24.2024.d1 Extended Data Python code implementing the simulation and analysis Download Extended Data, ZIP file.

## Results

### The virtual aging brain model replicates the age-related changes of sleep slow waves

Simulated SW characteristics along the virtual aging trajectory (reduction in inter-hemispheric connections) show the same trends of changes as the reported empirical observations. In particular, with age, the frequency of the SW decreases (Fig. 2*a*), the coefficient of variation increases (Fig. 2*d*), and the amplitude of the SW decreases (Fig. 2*e*). The shape of the SW also changes, namely the down-states become longer with age (Fig. 2*c*) while up-states remain almost unchanged (Fig. 2*b*). All panels of Figure 2 show the variability of the average channel characteristics of the SW across 16 different realizations of the stochastic process. We refer to Extended Data Figure 2-1 for the parameter differences assessed with Kruskal–Wallis for each degradation level *α*.

10.1523/ENEURO.0180-24.2024.f2-2 Figure 2-2 Download Figure 2-2, TIF file.

**Figure 3.**
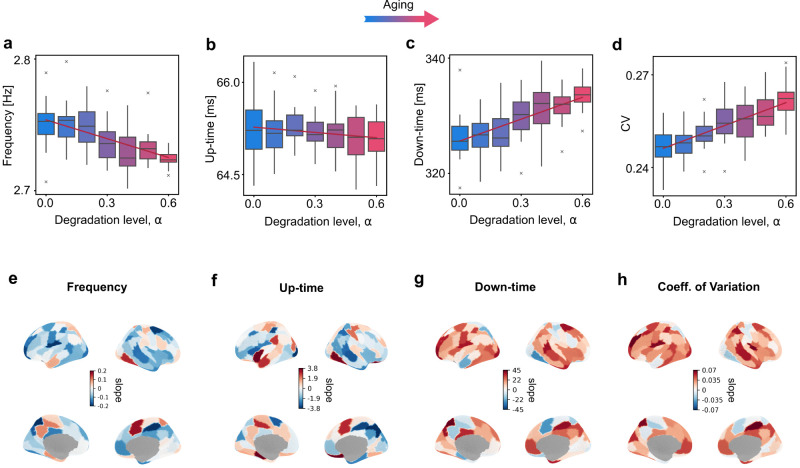
Relationship between degradation level (increasing synthetic aging) and slow wave oscillation characteristics across all nodes (first row) and at a single-node level (second row) for frequencies ***a, e***, (Freq.) average up–***b, f***, and down-state duration ***c, g***, and coefficient of variation ***d, h***, (CV). In the first row ***a-d***, each box-and-whisker plot represents the distribution of the considered value (e.g., frequency) across 16 realizations of noise averaged over all nodes, obtained for each single weight used to scale the structural connectivity matrix on 30 s simulations. The red line in each graph represents the linear fit across the mean values for each synthetic age (e.g., each weight). The second row ***e-h***, shows a brain surface—for the 100 regions of the Schaefer atlas—colored proportionally to the slopes of change of each single node, showing if the considered value (e.g., Frequency) increases (positive values) or decreases (negative values) along the synthetic aging process for that node. Extended Data Figure 3-1 details the robustness of the results with respect to the choice of the up-state threshold. Extended Data Figure 3-2 shows the complementary effect of the additional deterioration of the fronto-parietal tracts. Extended Data Figure 3-3 shows the location of the frontal and parietal areas.

10.1523/ENEURO.0180-24.2024.f2-3 Figure 2-3 Download Figure 2-3, TIF file.

To simulate other reported effects of the aging process (Coelho et al., 2021; Petkoski et al., 2023), particularly affecting the fronto-parietal connections, the left fronto-parietal connections (Extended Data Fig. 3-3, lobes defined by FSL MNI atlas Collins et al., 1995) were reduced similarly as described in Section 3.1, with the degree of connectome degradation represented by the parameter *β* ∈ [0.1, 0.6]. The results, reported in the Extended Data Figure 3-2, well align with those in Figure 3, showing that the additional decrease in connectivity further amplifies the effects observed after the reduction of inter-hemispheric connections.

10.1523/ENEURO.0180-24.2024.f3-1 Figure 3-1 Download Figure 3-1, TIF file.

10.1523/ENEURO.0180-24.2024.f3-2 Figure 3-2 Download Figure 3-2, TIF file.

10.1523/ENEURO.0180-24.2024.f3-3 Figure 3-3 Download Figure 3-3, TIF file.

### Network changes have spatially differential impact

**Figure 4.**
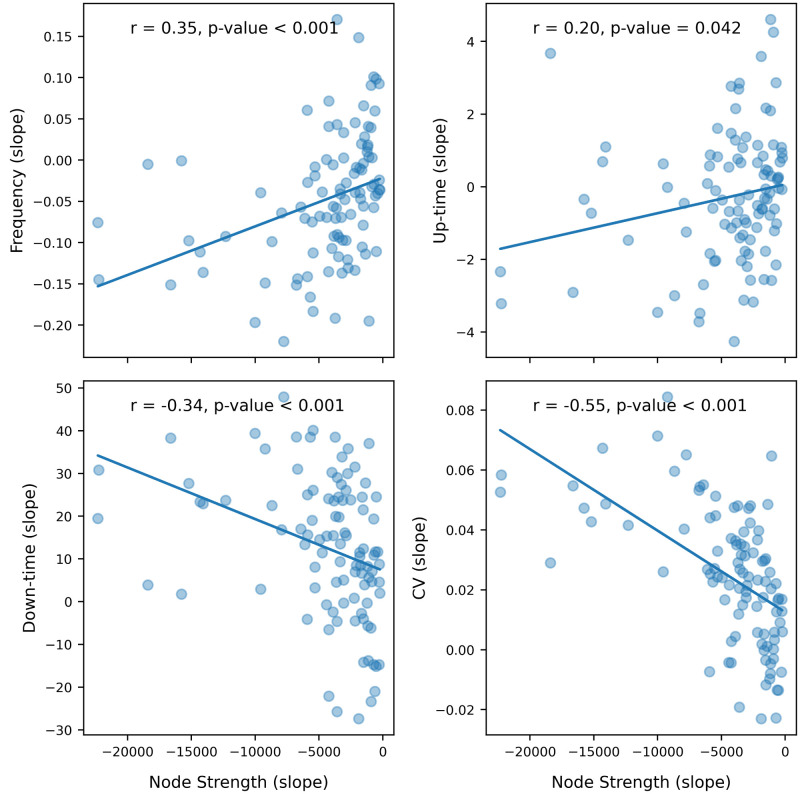
Regional association between rates of change (slope) in SW features and rates of change (slope) in graph theory metrics–node strength–across different levels of synthetic aging for each node of the 100-Schaefer atlas. The slope in all cases is the rate of change of the given metric over the levels of synthetic aging. Each point represents an average value across different noise realizations. Extended Figure 4-1 shows the rate of change of the SW features with respect to eigenvector centrality. Extended Figure 4-2 provides an overview of which nodes initiate the slow waves across the ageing trajectory. Extended Figure 4-3 shows the summary statistic for the SW initiation.

The computational model provides access to the activity on the level of individual nodes in addition to the simulated EEG. There, the age-related trends of the main characteristics of the EEG slow oscillations described in the previous section were preserved when averaged over the nodes of the network: the frequency decreased (Fig. 3*a*) and the coefficient of variability increased (Fig. 3*d*). In agreement with the spiking neural network (El Kanbi et al., 2023), the duration of the down state increased while the duration of the up state slightly decreased (Fig. 3*b*,*c*). At a node-specific level, instead, different nodes exhibited different rates of change of the SW characteristics along the virtual aging trajectory (Fig. 3*e*–*h*). When evaluating the linear relationship between nodal SW characteristics and alterations in graph properties of the SC along the virtual aging trajectory (Fig. 4 and Extended Data Fig. 4-1), we observed a reduction in nodal frequency and up-time duration with diminishing nodal strengths, and an increase in nodal down-time and CV with reducing nodal strengths. Lastly, we explored whether there was a change across the aging trajectory in terms of initiation of the individual slow waves–that is which nodes arrive first to the up-state within the synchronous wave. While certain nodes initiated the slow wave more frequently than others, this didn’t change significantly across the ageing trajectory (Extended Data Figs. 4-2 and 4-3).

10.1523/ENEURO.0180-24.2024.f4-1 Figure 4-1 Download Figure 4-1, TIF file.

10.1523/ENEURO.0180-24.2024.f4-2 Figure 4-2 Download Figure 4-2, TIF file.

10.1523/ENEURO.0180-24.2024.f4-3 Figure 4-3 Download Figure 4-3, TIF file.

### Decrease in EEG amplitude reflects decrease in inter-region synchrony

**Figure 5.**
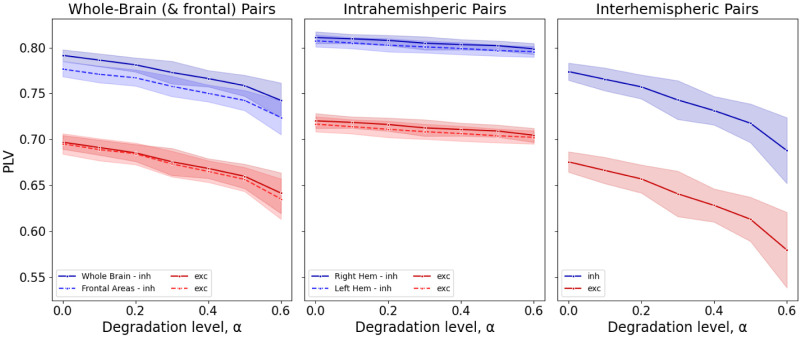
Mean PLVs, averaged over all trials (n = 15), as a metric for phase synchronization between the different nodes. Increasing degradation level denote virtual aging, which is characterized by a decrease in synchrony. The observed decrease of synchrony in the level of whole-brain ***a***, is driven by a decrease in the interhemispheric synchrony ***c***, as a minimal effect is detected in the synchrony within the same hemisphere ***b***. The frontal areas (*a*—dotted line) reflect the same tendency in synchrony as the whole brain. Extended Figure 5-1 shows the selected frontal areas and their connections. Extended Figure 5-2 shows an example time-course of the SW dynamics.

The decrease in amplitude is observed both in the empirical EEG data (El Kanbi et al., 2023; Fig. 1*d*) and in our simulated EEG data (Fig. 2*e*). However, no such decrease was observed on the network level. In order to explain this discrepancy, we turned to synchrony on the network level as the desynchronized oscillations would translate to smaller amplitudes on the EEG level due to linear mixing of the network-level signals through the leadfield matrix (Section 3.3.1). Indeed, the synchrony between the nodes, quantified by the PLVs, decreased as a function of increased virtual aging (Fig. 5*a*) for both excitatory and inhibitory neural populations. Moreover, this decrease was driven by the decrease of synchrony between inter-hemispheric pairs of nodes (Fig. 5*b*,*c*).

10.1523/ENEURO.0180-24.2024.f5-1 Figure 5-1 Download Figure 5-1, TIF file.

10.1523/ENEURO.0180-24.2024.f5-2 Figure 5-2 Download Figure 5-2, TIF file.

## Discussion

In this study, we aimed to investigate whether alterations in SW characteristics observed with aging derive from a reduction in nodal excitatory drives due to diminished inter-hemispheric connectivity. To assess this, we simulated SW oscillations by using a mean field whole-brain network model of sleep-like activity (AdEx) (Zerlaut et al., 2018; Di Volo et al., 2019; Goldman et al., 2022), where nodal activities were coupled by an underlying structural connectivity matrix. We employed the “virtual-aging” framework (Lavanga et al., 2023a), and, starting from a representative structural connectome (at the beginning of the aging trajectory), we synthetically reduced only the inter-hemispheric connections to simulate aging.

First, we were able to qualitatively reproduce, in our simulations, the age-related changes in SW characteristics observed in real empirical EEG recordings (El Kanbi et al., 2023). Notably, the simulated SW successfully mirrored the decreased SW frequency and duration of Up-states, increased variability and prolonged duration of Down-states observed at the EEG-level with aging (Van Cauter et al., 2000; Landolt and Borbély, 2001; El Kanbi et al., 2023). Similar effects were observed for slow-waves in an early-aging model in mice (Castano-Prat et al., 2017). Inter-hemispheric connections are well-known to decrease with aging (Puxeddu et al., 2020; Lavanga et al., 2023a) and our results align with previous literature showing their importance in influencing both awake resting state activity (Lavanga et al., 2023a) and sleep SW characteristics (Avvenuti et al., 2020). Previous work has hypothesized that a reduction in the neural cortical excitatory drive underlies many of the SW alterations observed with aging (Castano-Prat et al., 2017; El Kanbi et al., 2023). In particular, both electrophysiological findings on a mouse model of aging (Castano-Prat et al., 2017) and simulations from a computational model (El Kanbi et al., 2023) suggested that slower speed of propagation and diminished frequency of SW were linked to reduced cortical excitability. Here, by characterizing inter-hemispheric connections as only excitatory, we were able to provide evidence for the network origin of this reduced excitatory drive. In fact, diminishing inter-hemispheric connections in the model is equivalent to a reduction in the external excitatory drive for each connected node. Even though the nodes of the network were identical in their mean field model parameters, the effect of the reduced interhemispheric connections was spatially heterogeneous. Such heterogeneous age-related changes in structure-function coupling have been previously reported in awake fMRI data (Zamani Esfahlani et al., 2022), and together with our results suggest an intriguing direction for future empirical cohort studies of sleep EEG data. Given that previous studies have reported that the effects of aging also include the reduction of certain specific intra-hemispheric connections, we also investigated changes in slow-wave characteristics by selectively reducing the left fronto-parietal connections (Coelho et al., 2021; Petkoski et al., 2023). By applying the same “virtual aging” procedure, we showed that these alterations only strengthen the observed effects determined by the reductions in inter-hemispheric connections.

In empirical data, aging is also consistently linked with a decline in EEG amplitude (El Kanbi et al., 2023), suggesting a potential reduction in synchrony among brain regions. Notably, our virtual aging simulations revealed a more pronounced reduction in inter-hemispheric synchrony compared to intra-hemispheric synchrony. We thus suggest that reduced inter-hemispheric structural connections might also yield reduced inter-hemispheric synchrony and reduced EEG amplitude. SW are commonly conceptualized as traveling waves (Massimini et al., 2004), with propagation mainly via cortico-cortical white matter connections. Our results align with previous studies that demonstrated a direct association between parameters reflecting slow-wave synchronization and the microstructure (Buchmann et al., 2011; Piantoni et al., 2013) and structural integrity (Avvenuti et al., 2020) of the corpus callosum. In this view, inter-hemispheric connections likely serve as the primary pathway for cross-hemispheric propagation of slow waves (Avvenuti et al., 2020). Furthermore, the loss of slow-wave synchrony may further impact the high-frequency activity related to plastic processes such as memory consolidation, which is modulated by the slow-wave activity and potentially relying on the synchronous slow-waves for efficient inter-area communication (Helfrich et al., 2018; Staresina et al., 2023).

It should be noted that our simulations were performed only on one representative connectome, drawn from the embedding of connectomes of adults aged 55–63, that was “virtually aged.” Thus, future studies could aim at extending our results to empirical cohorts in order to address the inter-individual variability. In particular, virtual brain models with model parameters personalized with respect to individual EEG data would allow for relating the structural and functional changes to the decline in cognitive performance and other factors (Lavanga et al., 2023a). While the starting point of the virtual aging trajectory falls into the middle-aged adult bracket, it reflects the starting point of both the white matter changes (Schilling et al., 2022), and changes in the slow wave characteristics of the sleep (Mander et al., 2017; El Kanbi et al., 2023).

Furthermore, in this study, we only focused on inter-hemispheric connectivity, thus we cannot exclude that other factors known to be associated with aging (e.g., age-related neuromodulatory changes or grey matter atrophy) might also play a role in shaping SW characteristics. In particular, the role of aging-associated cholinergic reduction (Muir, 1997), which can be modeled as a variation of the adaptation parameter of the AdEx model (Brette and Gerstner, 2005; Zerlaut et al., 2018), should be further explored in future studies. While the local and network contributions to the reduced excitatory drive are difficult to disentangle, the respective hypotheses can be implemented in the virtual model of the mouse brain and augment the interpretation of the data on age-related changes of the slow wave characteristics of brain activity in animal models of aging (Melozzi et al., 2017; Sacha et al., 2024), including interventional studies (Spiegler et al., 2020).

The understanding of the age-related changes in sleep is important also due to sleep being implied as an intervention target in the neurodegenerative diseases (Tatulian, 2022). In fact, the disease can accelerate the age-related changes of the sleep processes reflected in the EEG features, such as the decrease in amplitude of the slow wave power (for review see Romanella et al., 2021). Moreover, the mechanistic models have recently provided explanations of the characteristic frequency slowing of the spontaneous awake brain activity in terms of local changes of the neural mass parameters due to the neurotoxicity (Alexandersen et al., 2023; Cabrera-Àlvarez et al., 2024). Integrating these models with the model of the aging sleep brain state presented in this paper constitutes an intriguing area of future work.

## Conclusion

In conclusion, our study sheds light on the potential role of inter-hemispheric connectivity in shaping age-related changes in SW sleep. By bridging the gap between mechanistic modeling and empirical observations, our findings pave the way for further research aimed at understanding and improving sleep quality in the aging population.

## Synthesis

Reviewing Editor: Arvind Kumar, KTH Royal Institute of Technology Decisions are customarily a result of the Reviewing Editor and the peer reviewers coming together and discussing their recommendations until a consensus is reached. When revisions are invited, a fact-based synthesis statement explaining their decision and outlining what is needed to prepare a revision will be listed below. The following reviewer(s) agreed to reveal their identity: Thomas Hinault.

Synthesis

The two reviewers thought that the manuscript addresses an important question - how aging affects slow wave sleep. The reviewers however think that the way 'virtual aging' is implemented is not quite what happens in 'actual aging'. While interhemispheric connectivity is weakened, other parts of the structural connectivity are also affected - for instance the fronto-parietal tracts. Therefore we think that the model has missed out on some crucial data on aging related changes in the structural connectivity. So we would suggest including other structural changes in the model and also simulate virtual aging in a more realistic manner. The comments from the reviewers are appended below to help you revise the manuscript according to their suggestions.

Reviewer #1

The manuscript "A whole-brain model of the aging brain during slow-wave sleep" uses a whole-brain model of adaptive exponential IF neurons to explore the dynamics of slow-wave sleep during aging. The manuscript is exceptionally well-written and demonstrates that the degradation of inter-hemispheric connections could be responsible for the aging symptoms of slow-wave sleep. I happily suggest that this manuscript be accepted after some minor clarifications of the contents of the Results. See the comments below.

Specific comments

In Section 3.4.2, could you clarify further how the frequencies were computed from the simulated data?

In Line 170, there is incorrect spacing after the equation for the PLV

In Figure 2, where does the standard deviation come from? Are they repeated simulations where the stochasticity comes from the OU process alone, or are there other differences between them? Is the figure showing the means across the nodes? Are they computed via the virtual electrodes or the model variables directly? It would be easier for the reader to have this clarified in Section 4.1 and the figure caption.

Lines 205-207: Can you clarify what "frequency of slow-wave initiation" means?

In Figure 4, are these data points from different points of aging, or are they averaged over aging? Are all the simulated data points over repeated simulations and over different age points lumped together? Please clarify this point.

Section 4.3 mentions a decrease in amplitude in simulations. However, this decrease is not shown in the figures or reported in the text (please correct me if I am wrong). The decrease in amplitude should be shown, or the sentence should be rewritten. That the simulations spend more time in the "down-state" does not necessarily imply that the amplitudes decrease (the waves could potentially spend more time in the through than the peak but still have the same amplitude).

In the context of neurodegenerative disease, whole-brain models have also been used to study frequency slowing due to evolving connectomes (see papers below), with reduced excitability also being the culprit. This could be worth mentioning in the Discussion

https://royalsocietypublishing.org/doi/full/10.1098/rsif.2022.0607

https://pubmed.ncbi.nlm.nih.gov/38565295/

Reviewer #2

This study investigated aging effects on sleep slow-waves using a whole-brain computational model. The association between structural connectivity changes (mainly inter-hemispheric connectivity) and resting-state dynamics was the focus of the modelling approach. Results reveal that the model could account for shorter and for variable slow waves with aging, in association with structural changes.

Overall, the paper is well written and investigates an important research question. The modelling approach is ideally suited to test mechanistic assumptions about brain aging. However, important theoretical and methodological clarifications are necessary. Below are some comments that could assist the authors in refining the manuscript.

Major points

1. My main concern is about the current focus on inter-hemispheric structural and functional connectivity. While I do not deny these changes, aging effects on structural connectivity are far from restricted to these connections, as extensive changes have also been reported in longer, fronto-parietal tracts (e.g., Coelho et al., 2021, Scientific Reports; Petkovski et al., 2023, Cerebral Cortex). This has been the focus of several wake EEG work (see Courtney &Hinault, 2021, Progress in Neurobiology, for a review). I would recommend including these changes in the model to provide a more accurate account of structural changes during aging.

2. Furthermore, the current approach appears to be more adapted to model brain lesions and pathological changes than healthy aging. Indeed, the Methods section (p.3) indicates the "elimination of inter-hemispheric connections". This is not what occurs in healthy aging and certainly not at the age range considered (55-63 old). Physiological aging is rather characterized by slight degradations of white matter integrity leading to noisier and delayed functional communications. This strongly differs from the disconnection induced by the complete interruption of structural connections.

3. Relatedly, the paper should account for the potential limitation of the inclusion of very young individuals (55-63 old), which given the overall increase of the human lifespan and of the retirement age is far from reflecting changes occurring during the third tier of life.

4. Can the model account for different age-related trajectories? One could expect the age-related variability to be associated with lower cognitive reserve or white matter integrity, with subgroup differences relative to individuals with preserved integrity levels.

5. Relative to PLV, phase-lag index (PLI) is less susceptible to volume conduction artefacts (e.g., Li et al., 2021, Cognitive Neurodynamics). These could have influenced the reported results.

Minor points

1. Several methodological decisions (notably sections 3.4.1 and 3.4.2) should be further justified and documented.

2. Additional details should be provided regarding source modelling.

3. The Discussion section should be further developed, notably regarding the loss of synchrony in functional communications and for implications of the reported results for models of brain aging.
