# Supplementary figures and images for: A Whole-Brain Model of the Aging Brain During Slow Wave Sleep
Source: eNeuro. 2024 Nov 5;11(11):ENEURO.0180-24.2024. doi: 10.1523/ENEURO.0180-24.2024 (PMC11540593; doi:10.1523/ENEURO.0180-24.2024)

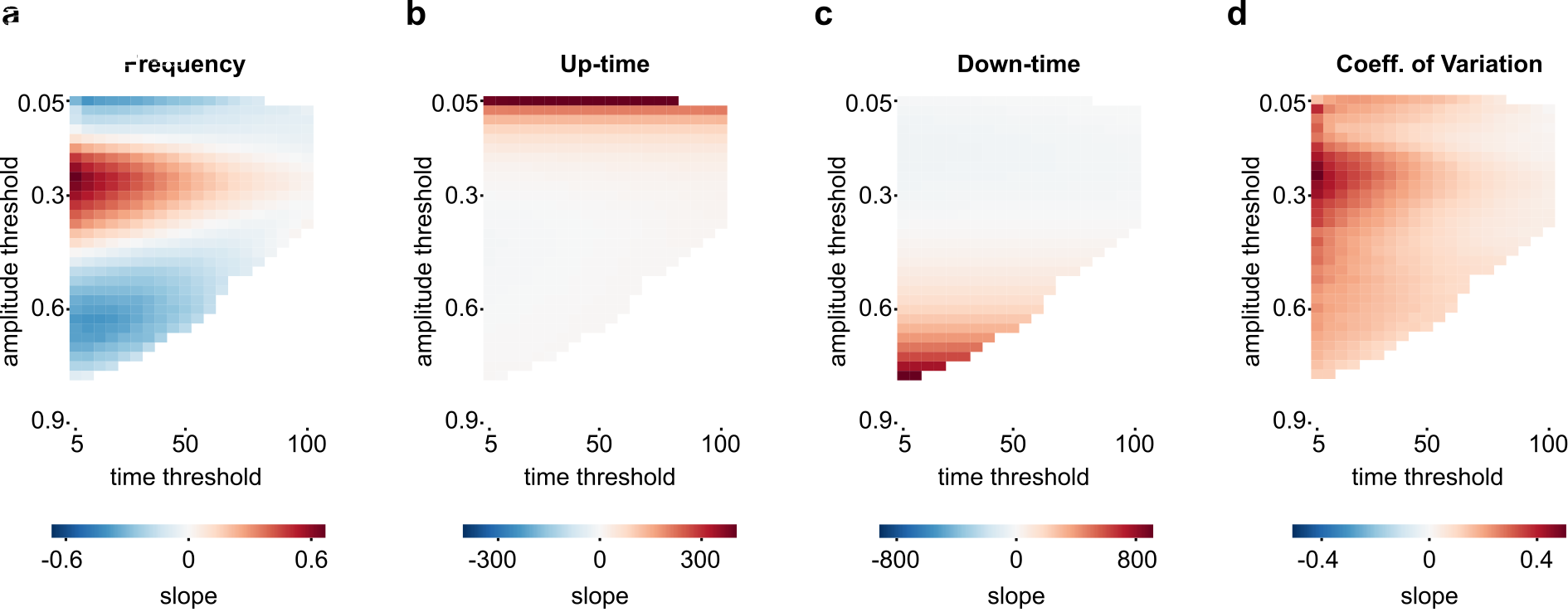

Supplement: Figure 2-2 — Download Figure 2-2, TIF file. [file eneuro-11-ENEURO.0180-24.2024-s013.tif]

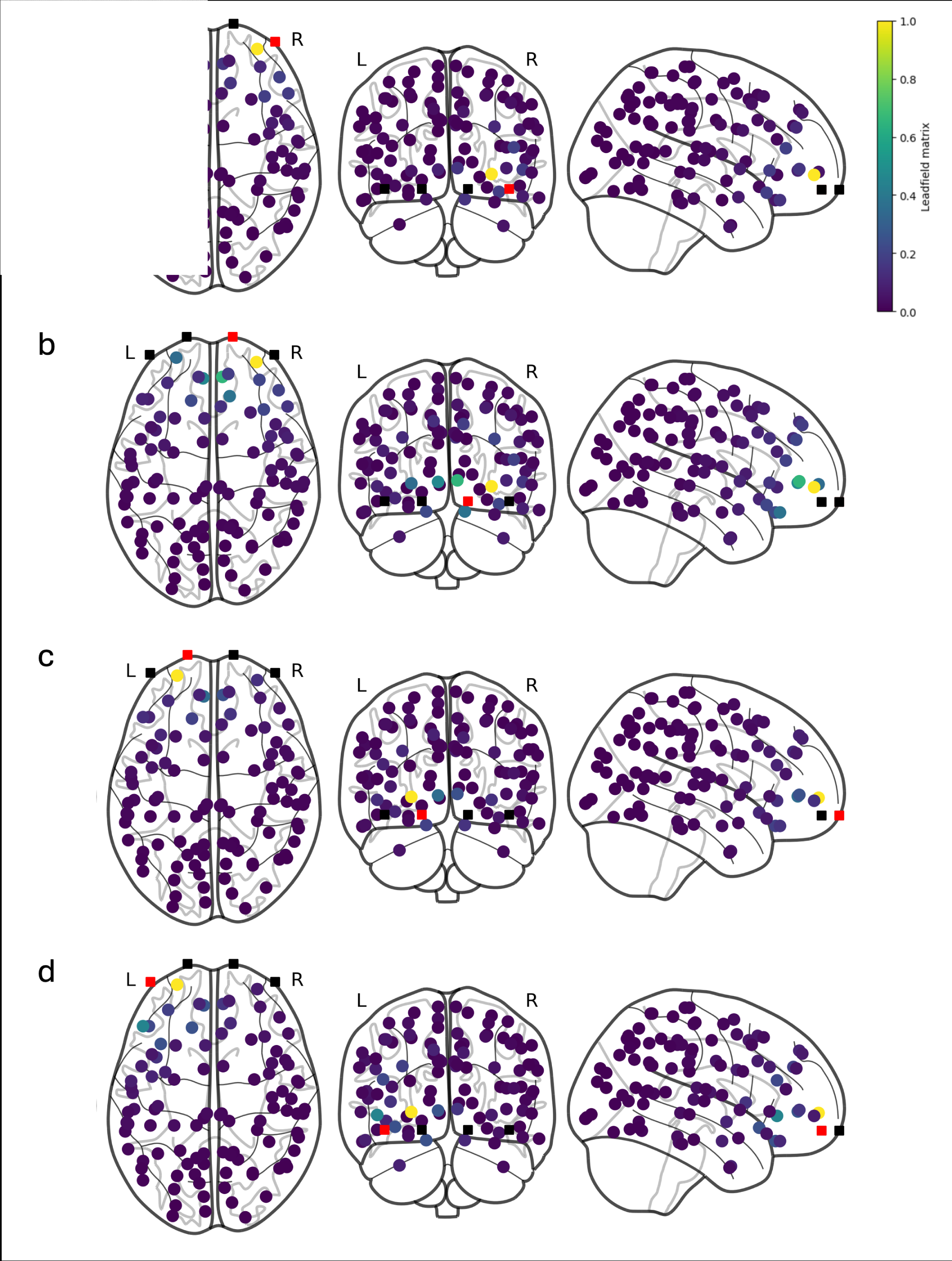

Supplement: Figure 2-3 — Download Figure 2-3, TIF file. [file eneuro-11-ENEURO.0180-24.2024-s015.tif]

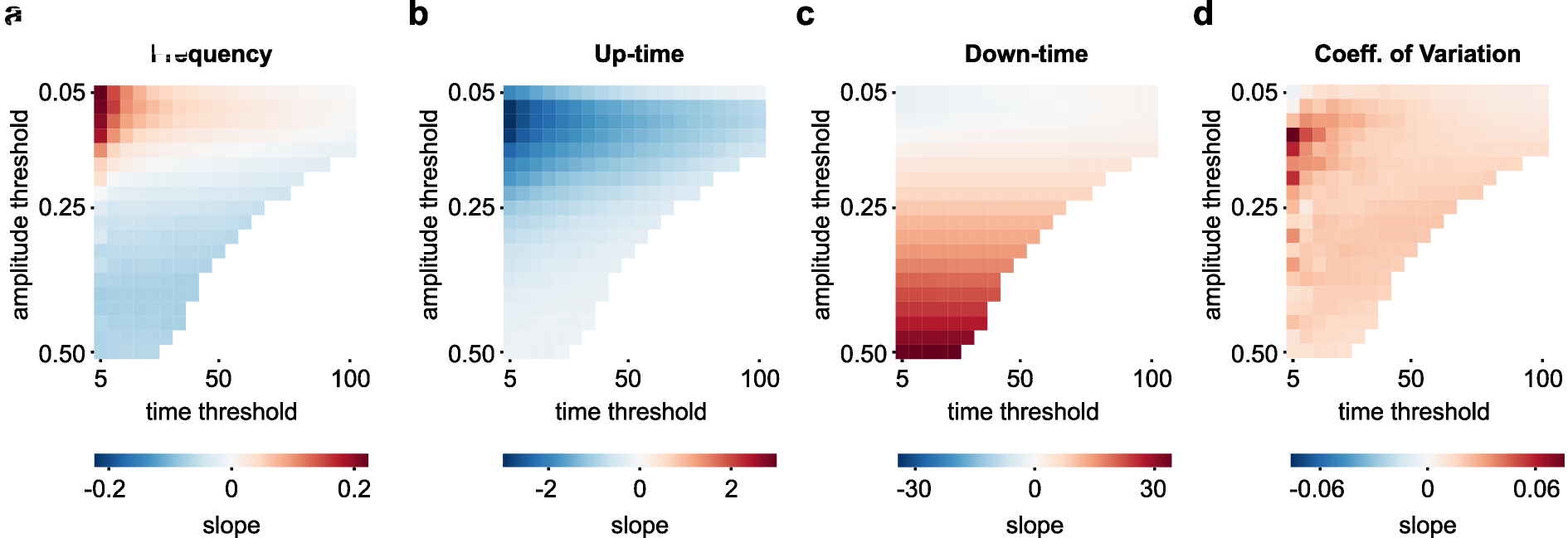

Supplement: Figure 3-1 — Download Figure 3-1, TIF file. [file eneuro-11-ENEURO.0180-24.2024-s014.tif]

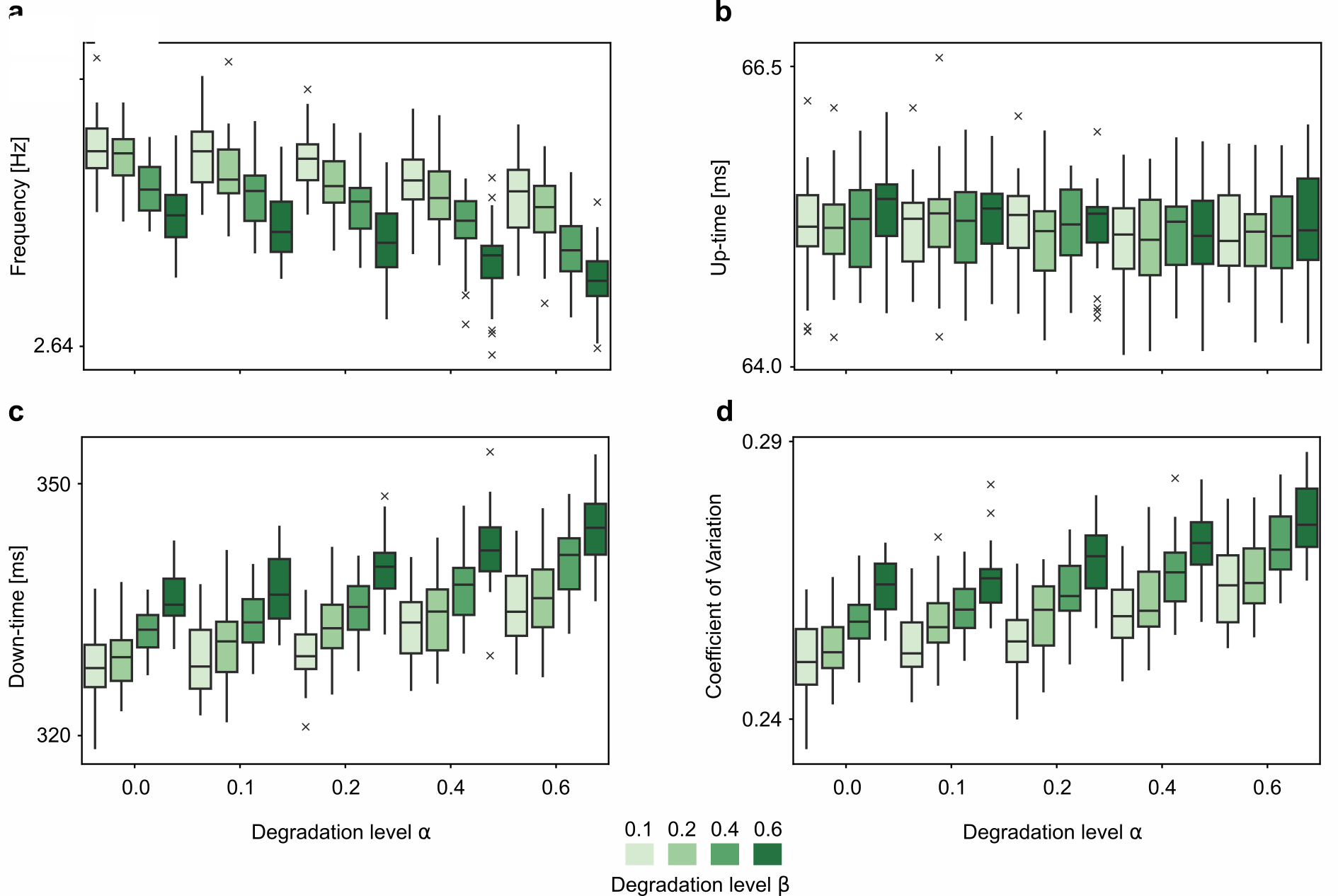

Supplement: Figure 3-2 — Download Figure 3-2, TIF file. [file eneuro-11-ENEURO.0180-24.2024-s008.tif]

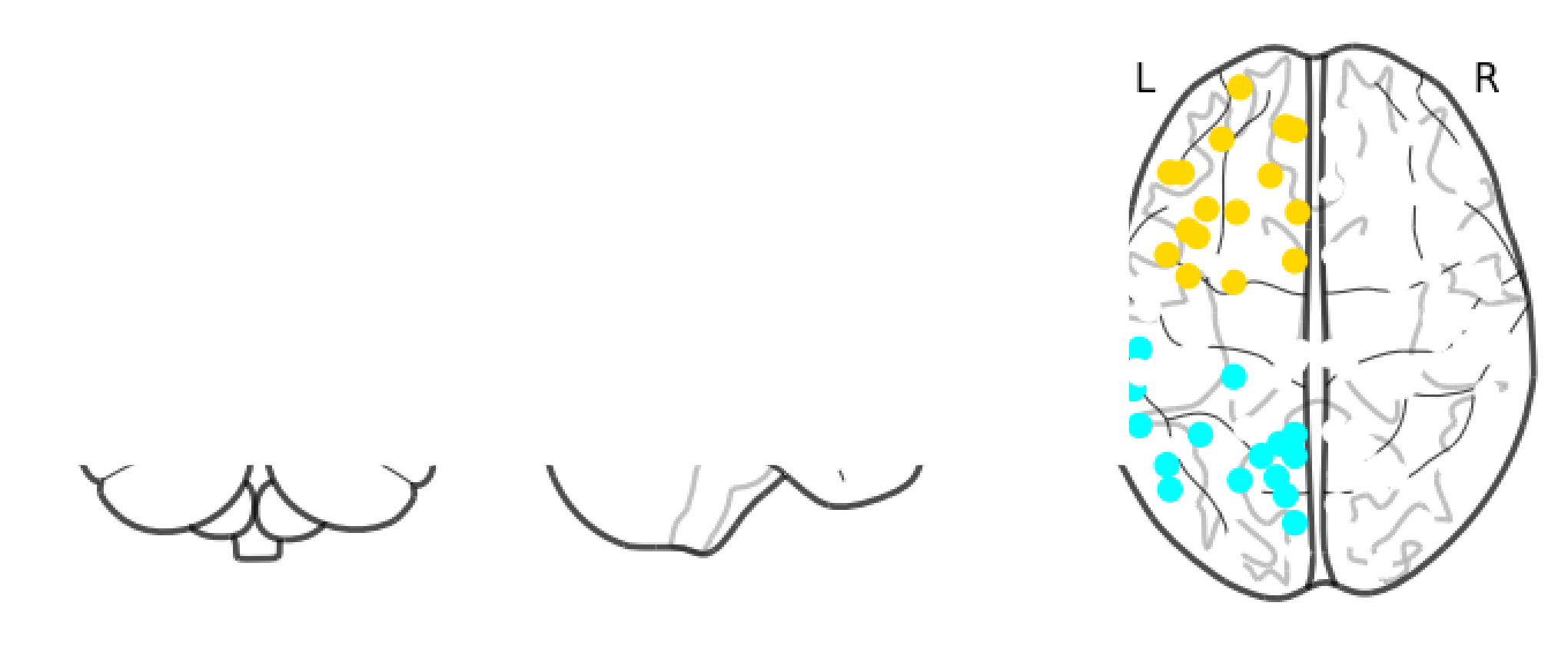

Supplement: Figure 3-3 — Download Figure 3-3, TIF file. [file eneuro-11-ENEURO.0180-24.2024-s011.tif]

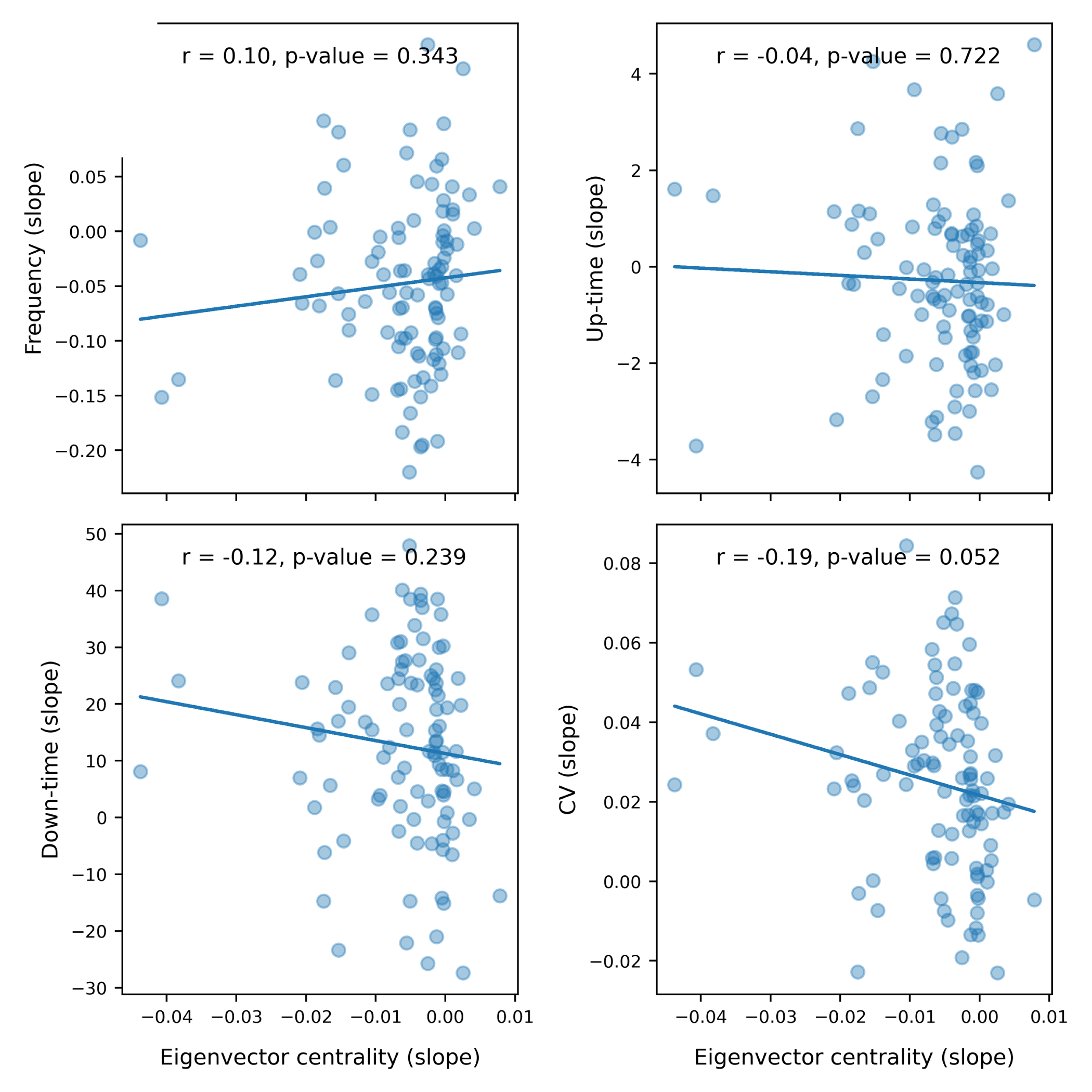

Supplement: Figure 4-1 — Download Figure 4-1, TIF file. [file eneuro-11-ENEURO.0180-24.2024-s012.tif]

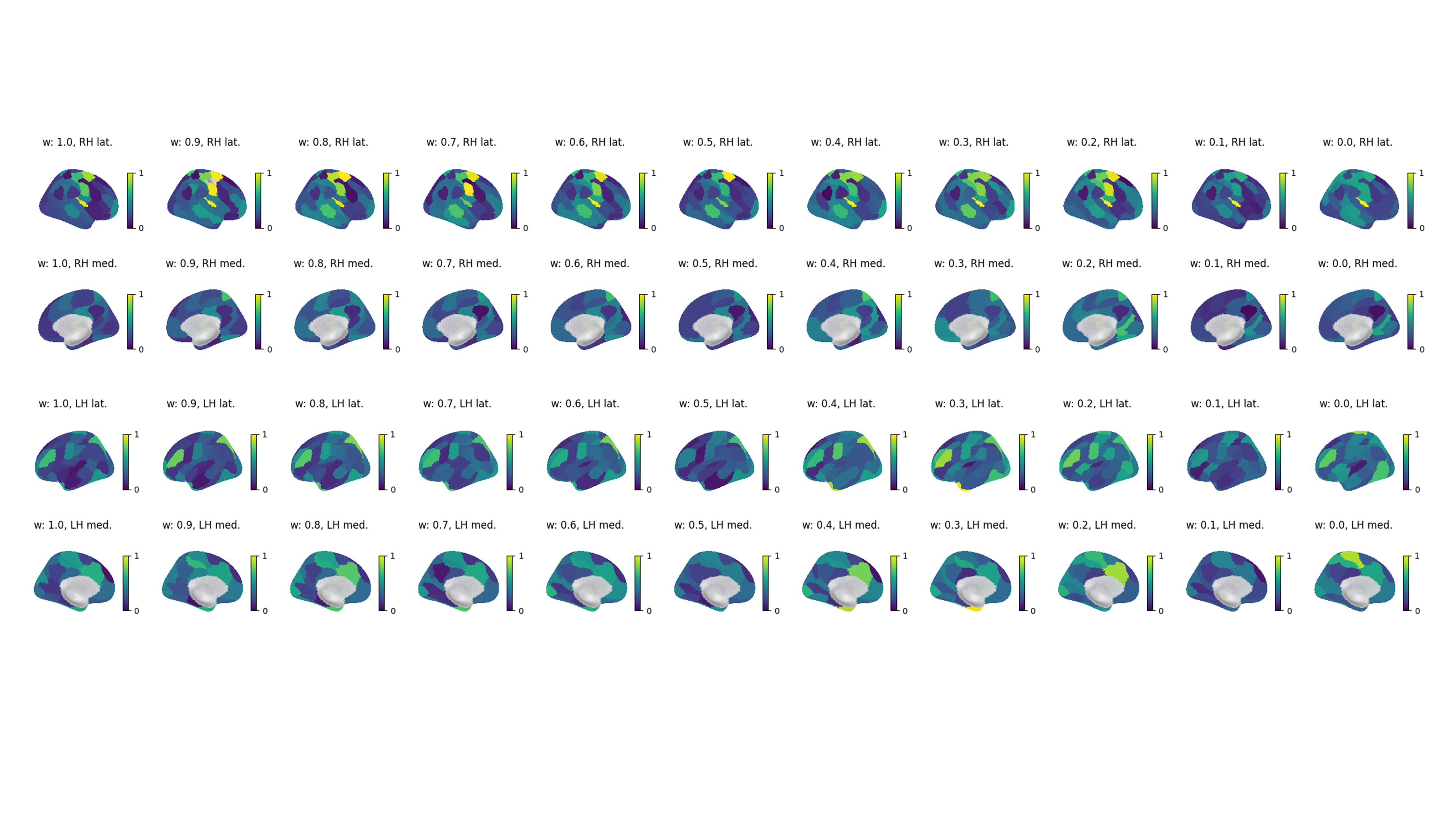

Supplement: Figure 4-2 — Download Figure 4-2, TIF file. [file eneuro-11-ENEURO.0180-24.2024-s010.tif]

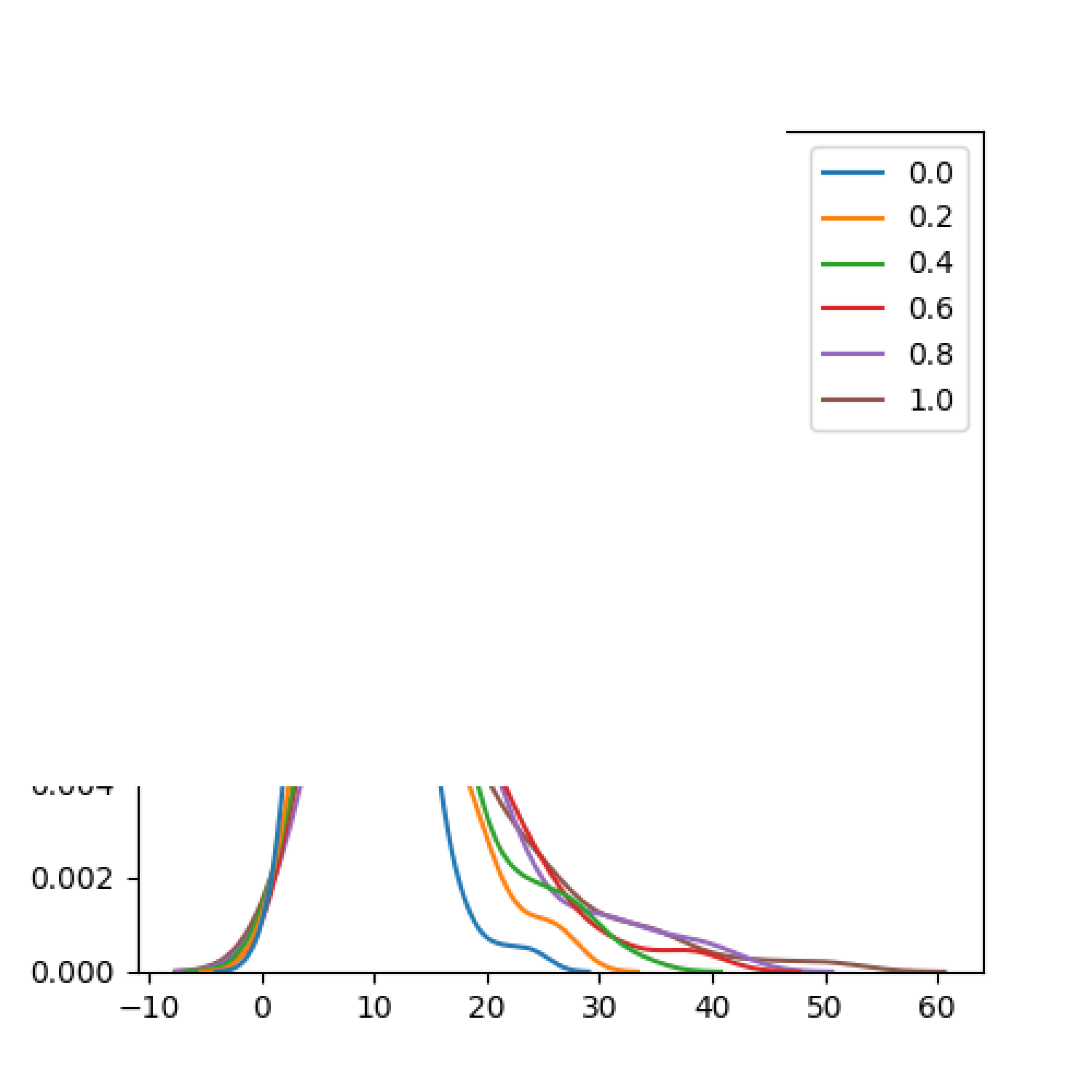

Supplement: Figure 4-3 — Download Figure 4-3, TIF file. [file eneuro-11-ENEURO.0180-24.2024-s009.tif]

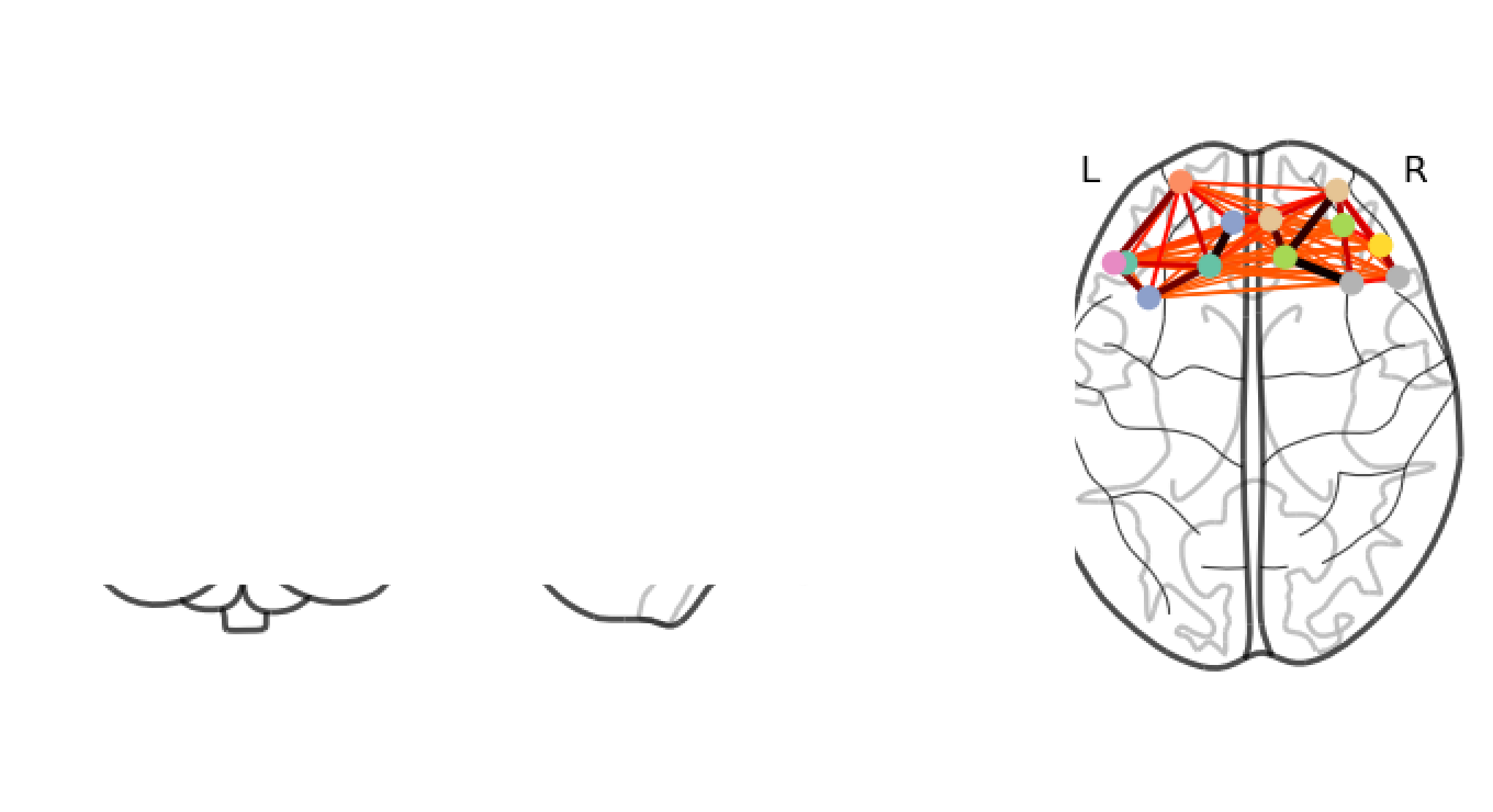

Supplement: Figure 5-1 — Download Figure 5-1, TIF file. [file eneuro-11-ENEURO.0180-24.2024-s007.tif]

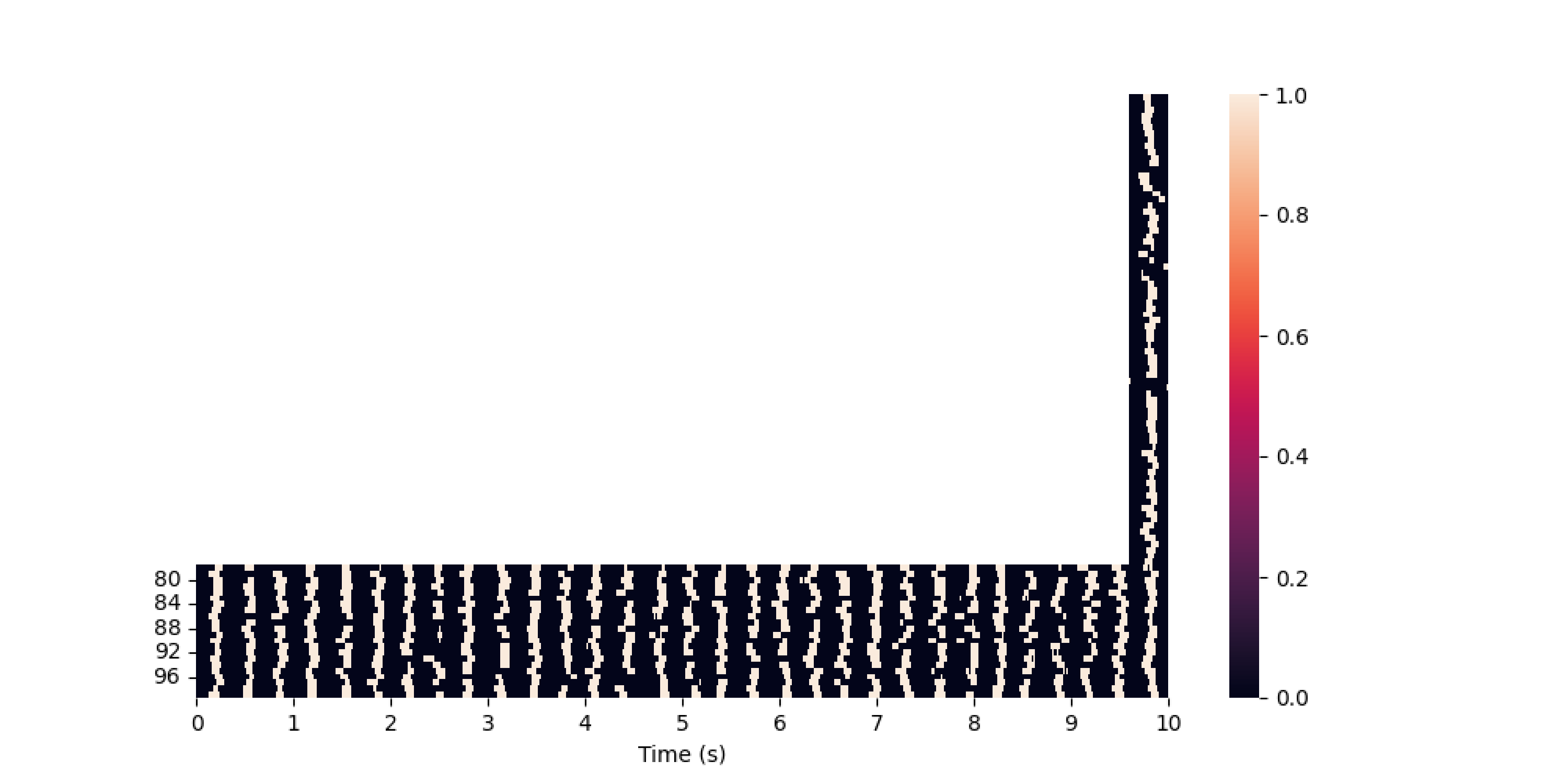

Supplement: Figure 5-2 — Download Figure 5-2, TIF file. [file eneuro-11-ENEURO.0180-24.2024-s006.tif]
